# The Relationship between Symptoms and Social Functioning over the Course of Cognitive Behavioral Therapy for Social Anxiety Disorder

**DOI:** 10.1155/2020/3186450

**Published:** 2020-09-28

**Authors:** Sei Ogawa, Risa Imai, Masako Suzuki, Toshi A. Furukawa, Tatsuo Akechi

**Affiliations:** ^1^Graduate School of Humanities and Social Sciences, Nagoya City University, Nagoya, Japan; ^2^Department of Psychiatry and Cognitive-Behavioral Medicine, Nagoya City University Graduate School of Medical Sciences, Nagoya, Japan; ^3^Department of Health Promotion and Human Behavior, Kyoto University Graduate School of Medicine/School of Public Health, Kyoto, Japan

## Abstract

**Background:**

The present study is aimed at investigating the relationship between changes in symptoms and changes in social functioning during cognitive behavioral therapy (CBT) for social anxiety disorder (SAD).

**Methods:**

Ninety-six patients with SAD were treated with manualized group CBT. Measures of social anxiety symptoms, depression symptoms, cognition, and social functioning were administered at baseline and endpoint. Using multiple regression analysis, we examined the associations between the changes in four aspects (work, home management, social leisure activities, and private leisure activities) of social functioning as dependent variables and the changes in four factors (social interaction, public speaking, observation by others, and eating and drinking in public) in social anxiety symptoms, depression symptoms, and cognition as independent variables.

**Results:**

The changes in work functioning were predicted by the changes in the public speaking factor in social anxiety symptoms. The changes in depression symptoms predicted the changes in home management. The significant predictors of changes in social leisure activities were the changes in the social interaction factor and depression symptoms. The changes in private leisure activities were predicted by the changes in the observation by others factor. The changes in cognition predicted nothing.

**Conclusion:**

The present study suggested that the changes in social anxiety or depression symptoms may predict several aspects of social functioning changes in patients with SAD over the course of CBT. In order to improve social functioning, our results may be useful for selecting the fear or feared situation in CBT for SAD. *Trial Registration*. The clinical study registration number in the Japanese trials registry is UMIN CTR 000031147.

## 1. Background

Social anxiety disorder (SAD) is a serious disabling disease associated with remarkable impairment in quality of life (QOL) or social functioning [1]. Patients with SAD are significantly impaired in social functioning compared with the general population [2]. In addition, patients affected by SAD are more financially dependent [3], underemployed, and less productive at work compared with those without the disorder [4]. The impairment caused by SAD is very high, and SAD is among the five most impairing psychiatric disorders (dysthymia, major depressive episode, posttraumatic stress disorder, panic disorder, and SAD) [5]. Improvements in social functioning, as well as symptomatic relief, are important outcomes for the treatment of SAD.

A large number of randomized controlled trials support the use of cognitive behavioral therapy (CBT) for SAD [6]. Meta-analyses have concluded that there is strong empirical support for the efficacy of CBT for SAD [7]. Moreover, several studies mentioned that the effects of CBT may extend beyond a reduction in social anxiety symptoms to improvements in social functioning [8–10]. Although anxiety symptoms might respond to CBT for anxiety disorder in the short term, longer treatments seem to be needed to improve QOL or social functioning. In order to explain this difference, an analysis of the QOL or social functioning construct in CBT is required [11]. However, there are few studies in support of the specific mechanisms of change in social functioning during CBT.

The cognitive behavioral model is based upon the assumption that our cognition influences our behavior or emotions. CBT focuses on unhelpful cognition and behaviors and improves QOL or social functioning as mentioned. In the previous research of anxiety disorder including panic disorder, generalized anxiety disorder, and posttraumatic stress disorder, QOL may change as a result of, or simultaneously with, anxiety symptom changes, not cognitive changes [12]. For the purpose of improving QOL or social functioning in CBT for these disorders, it may be useful to focus on achieving symptom improvements in preference to cognitive changes. However, this study did not include SAD. Therefore, additional research is needed to clarify the mechanisms of changes in social functioning in CBT for SAD. Moreover, CBT for SAD is adjusted to particular fears or feared situations in daily life. These fears or situations are chosen based on what is treatable or the purpose of the treatment. It is important to select the fears or situations for the effective CBT for SAD. The previous study showed that patients with SAD experienced improved social functioning after CBT [13]. However, there is no study to examine the relationship between social fear symptoms and social functioning during CBT.

The present study is aimed at investigating how changes in social functioning related to changes in symptoms of SAD over the course of CBT.

## 2. Methods

This prospective and observational study enrolled consecutive SAD patients treated with CBT in our institute. This study was conducted as a single-arm and naturalistic study in a routine clinical setting.

### 2.1. Patients

One hundred eleven patients participated in the present study from July 2003 to June 2012. Some participants were referred from mental health professionals, and others sought treatment for SAD on their own. All of the patients met the criteria for SAD as the primary diagnosis according to the DSM-IV criteria [14], assessed by the Structured Clinical Interview for DSM-IV (SCID) [15]. All of them fulfilled the following criteria: (i) absence of cluster B personality disorder, (ii) absence of substance use disorder, (iii) no history of psychosis or bipolar disorder, and (iv) no previous CBT treatment. The main reason for exclusion was that participants with severe psychiatric disease could not attend the group CBT sessions regularly. Although no other additional structured psychotherapies were allowed during the CBT, there was no restriction on concurrent pharmacotherapy.

The participants provided their written informed consent after receiving a full explanation of the study's purpose and procedures.

### 2.2. Treatment

We followed the group CBT manual for SAD developed by Andrews et al. [16] and integrated the Clark and Wells model of SAD [17] into the manual. The therapists had group discussions once a month to check on therapist adherence to the CBT program and to plan for future sessions. The program consisted of sixteen weekly sessions by two therapists, who were psychiatrists or clinical psychologists with more than two years of clinical experience. Each session was scheduled to last 120 minutes. The program included (i) psychoeducation about SAD including the Clark and Wells cognitive behavioral model, (ii) behavioral experiments to examine the participant's catastrophic predictions, (iii) attention training to shift focus from unhelpful thoughts and feeling to the task or the external situation, (iv) cognitive restructuring to identify and dispute maladaptive thoughts, and (v) in vivo graded exposures to anxiety-provoking situations.

### 2.3. Measurements

The patients were assessed with an extensive questionnaire battery using observer-rated assessments and self-report questionnaires at baseline and endpoint in our institute. We assessed the Liebowitz Social Anxiety Scale (LSAS), the Brief Fear of Negative Evaluation Scale (BFNE), the Symptom Checklist-90 Revised (SCL-90-R), and the Work, Home Management, Social and Private Leisure Activities Scale (WHLS).

#### 2.3.1. LSAS

The LSAS is a widely used clinician-administered psychological scale for assessment of SAD [18]. It is a 24-item scale that is rated in terms of both fear (0-3 indicate none, mild, moderate, and severe, respectively) and avoidance (0-3 indicate never, occasionally, often, and usually, respectively) of performance situations and social interaction. Moreover, separate exploratory common factor analyses of the fear and avoidance ratings yielded four similar factors for each: (1) social interaction, (2) public speaking, (3) observation by others, and (4) eating and drinking in public [19]. The reliability and validity of the Japanese version have been established [20]. The clinicians in charge of the CBT program administered LSAS at baseline and endpoint.

#### 2.3.2. BFNE

The BFNE is a 12-item self-reported instrument developed to measure fear of negative evaluation in social situations [21]. Each item is rated on a 5-point scale of 0 (not at all characteristic of me) through 4 (extremely characteristic of me). This scale is a brief version of the original 30-item Fear of Negative Evaluation (FNE) scale [22]. The FNE assesses fear of negative evaluation by others, a core cognitive feature of SAD. Fear of negative evaluation is recognized in cognitive models of SAD [23]. Furthermore, there is substantial empirical evidence that fear of negative evaluation is a core cognitive feature of SAD [24]. The BFNE is more sensitive to treatment-related change than FNE [25]. Its Japanese version has been validated [26].

#### 2.3.3. SCL-90-R

The SCL-90-R is a 90-item symptom inventory for general psychopathology. The self-report questionnaire is divided into ten subscales of somatization, obsessive-compulsive, interpersonal sensitivity, depression, anxiety, hostility, phobic anxiety, paranoid ideation, psychoticism, and global severity index. Each item is scored on a five-point scale ranging from 0 (not at all) to 4 (extremely) [27]. The reliability and validity of the Japanese version have been shown [28]. The depression subscales have been often used as psychiatric outcome measures [29]. Therefore, we used the depression subscale including 13 items.

#### 2.3.4. WHLS

The WHLS is a self-report questionnaire for assessing functional impairments in the domains of work, home management, and social and private leisure activities. Each item is rated on a 9-point scale of 0 (not at all impaired) through 8 (very severely impaired). Satisfactory reliability and construct validity have been demonstrated [30]. The Japanese version has been validated [31].

### 2.4. Statistical Analyses

All the data were examined using the Statistical Package for the Social Sciences (SPSS) 18.0 for Windows [32]. The baseline and endpoint data were used to calculate change scores for each variable, denoted by delta (*Δ*). All the statistical tests were two-tailed, and results were considered statistically significant when the *p* value was less than 0.05. First, we compared the demographic and clinical data between the patients who completed the program and those who did not, using an independent-samples *t*-test or *χ*^2^ test. Second, we used a paired *t*-test to compare all the SAD symptomatology and social functioning scores between the pretreatment and the posttreatment. Third, simple correlations among change scores in SAD symptoms and cognitions were investigated to identify significant bivariate correlation. Fourth, to examine the predictors of changes in social functioning outcomes, we performed multiple linear regression analysis. We used the variables about the changes in the four factors of LSAS (social interaction, public speaking, observation by others, and eating and drinking in public), BFNE, and depression subscale of SCL-90-R as independent variables. The changes in the four subscales of WHLS (work, home management, social leisure activities, and private leisure activities) were used as dependent variables.

### 2.5. Ethical Approval

The study was performed in accordance with the Declaration of Helsinki, and the study's protocol was approved by the ethics committee of our institute.

## 3. Results

### 3.1. Patients

Among 111 patients who started the CBT program, 96 patients (86.5%) completed the treatment, even when they were absent from a few sessions. Fifteen patients dropped out prematurely from the treatment.  Table 1. shows the baseline demographic and clinical characteristics of the completers and the dropouts. No statistically significant differences were seen among the subgroups.

### 3.2. Pretreatment and Posttreatment Rating Scale Scores

Table 2. presents the pretreatment and posttreatment clinical rating scale scores. All the scores were improved significantly (*p* < 0.05).

### 3.3. Correlations among Change Scores

Bivariate correlations of change scores are shown in Table 3. There were significant correlations among changes in the depression subscale of SCL-90-R, BFNE, and four factors of LSAS. All of the correlations were less than 0.7 meaning that the correlation was relatively low and might not cause multicollinearity.

### 3.4. Predictors of the Outcome Changes

In multiple regression analysis, the reduction in the public speaking factor of LSAS predicted the reduction in the work subscale of WHLS (Table 4). The reduction in the home subscale was predicted by the reduction in the depression subscale of SCL-90-R (Table 5). The reduction in the social leisure activities subscale was predicted by the reduction in the depression subscale of SCL-90-R and the reduction in the social interaction factor of LSAS (Table 6). The reduction in the observation by others factor of LSAS predicted the private leisure activities subscale reduction (Table 7). The change in BFNE did not predict the changes in WHLS subscales.

The number of samples per an independent variable in a multivariate analysis should be more than ten [33]. In our study, the number of samples was 96 and we use six independent variables in multiple regression analysis. Therefore, the number of samples per one independent variable was 96/6 = 16. The sample size for this study was appropriate.

## 4. Discussion

The present study demonstrated that the changes in social anxiety or depression symptoms may predict several aspects of social functioning change in patients with SAD over the course of CBT, whereas the cognitive change was not related to the change in social functioning.

The present results are consistent with the finding of Oei et al. (2014) in CBT for other anxiety disorders that QOL change is related to anxiety and depression symptom changes. In patients with SAD, social functioning may be more closely linked to symptoms while changes in social functioning might occur independently of cognitive changes. Furthermore, Oei et al. (2014) showed that cognitive changes are not consistently associated with changes in QOL during CBT for other anxiety disorders.

The present study suggested that the mechanism of change in QOL or social functioning during CBT may be relevant to symptom changes rather than cognitive changes.

From a clinical point of view, our results suggest that it may be useful to focus on achieving symptom improvements in preference to cognitive change in order to improve social functioning during CBT for SAD.

A novel finding of our study is that the changes in factors in LSAS may predict the social functioning changes in CBT for SAD. Cognitive behavioral interventions are tailored to particular fears or feared situations in the patient's life. The situations may be chosen based on what is treatable or the patient's goal for treatment. For example, in order to improve especially working in social functioning, we could select the public speaking task in an exposure session. Our results may be useful for selecting the situation in tailored treatment. However, the adjusted *R*-squares in our results are relatively low. Further investigation is needed to clarify the factors influencing social functioning.

The present study has several limitations. First, this study was carried out as a naturalistic study and did not include a control group. Therefore, one cannot conclude if the significant changes in symptomatology and social functioning in the present cohort might be due to the passage of time rather than CBT for SAD. Second, antidepressant and benzodiazepine medications were permitted during CBT treatment. The information about the amount of drug use during the treatment was not collected. Hence, it is impossible to consider the effects of medications on CBT. Third, the clinicians in charge of the CBT program for SAD rated LSAS at baseline and endpoint. Accordingly, rating LSAS was not blind or independent. Fourth, the causality cannot be established between symptom changes and social functioning changes. Changes in social functioning may occur as a result of or simultaneously with symptom change. Fifth, this study lacked the data from every session and therefore we cannot analyze the mechanism of change over time during CBT. Sixth, the data was collected from 2003 to 2012 and considered to be rather old. However, the demographic and clinical characteristics of this cohort, including sex, mean age, onset, comorbidity, and LSAS total score, did not differ much from those of the general SAD population as shown in Table 1.

## 5. Conclusion and Recommendations

The present study suggested that the changes in social anxiety or depression symptoms may predict several aspects of social functioning changes in patients with SAD over the course of CBT. For the purpose of improving the social functioning, our results may be useful for selecting the fear or feared situation over the course of CBT for SAD.

## Figures and Tables

**Table 1:** Demographics and baseline characteristics, mean (SD) (*N* = 111).

Characteristics	Completers (*n* = 96)	Dropouts (*n* = 15)	*p*
Sex (% female)	52.1	46.7	0.79
Mean age	34.2 (10.8)	32.1 (11.9)	0.48
Onset	19.4 (8.1)	16.3 (5.1)	0.17
Current mood disorder (%)	17.7	20.0	0.83
Current panic disorder (%)	2.1	0	0.57
Current specific phobia (%)	2.1	0	0.57
SCL-90-R depression	1.36 (0.82)	1.53 (0.97)	0.46
BFNE	35.5 (10.1)	38.9 (9.0)	0.22
LSAS			
Total	75.5 (22.8)	85.3 (22.8)	0.17
Social interaction factor	38.8 (16.2)	44.4 (11.0)	0.10
Public speaking factor	21.3 (5.9)	21.9 (6.6)	0.69
Observation by others factor	11.2 (6.0)	13.7 (6.4)	0.26
Eating and drinking in public	4.2 (3.3)	5.3 (4.1)	0.26
WHLS			
Work	5.2 (2.1)	4.5 (1.8)	0.26
Home management	2.3 (2.0)	2.7 (2.0)	0.39
Social leisure activities	4.8 (2.2)	5.3 (2.1)	0.40
Private leisure activities	1.6 (2.0)	1.5 (2.1)	0.93

*p* values were calculated using *t* statistic for continuous variables and using *χ* statistic for categorical variables. SCL-90-R: Symptom Checklist-90 Revised; BFNE: Brief Fear of Negative Evaluation Scale; LSAS: Liebowitz Social Anxiety Scale; WHLS: Work, Home Management, Social and Private Leisure Activities Scale. Completers: the patients who completed the treatment, even when they were absent from a few sessions.

**Table 2:** Pretreatment and posttreatment rating scale scores, mean (SD) (*n* = 96).

	Pre-treatment	Post-treatment	*p*
SCL-90-R depression	1.36 (0.82)	0.97 (0.77)	<0.01
BFNE	35.5 (10.1)	27.7 (9.81)	<0.01
LSAS			
Total	75.5 (22.8)	55.8 (26.0)	<0.01
Social interaction	38.8 (16.2)	29.6 (15.0)	<0.01
Public speaking	21.3 (5.9)	15.6 (6.7)	<0.01
Observation by others	11.2 (6.0)	7.6 (5.1)	<0.01
Eating and drinking in public	4.2 (3.3)	3.0 (2.9)	<0.01
WHLS			
Work	5.2 (2.1)	3.6 (2.0)	<0.01
Home management	2.3 (2.0)	1.4 (1.6)	<0.01
Social leisure activities	4.8 (2.2)	3.2 (2.2)	<0.01
Private leisure activities	1.6 (2.0)	1.2 (1.7)	<0.05

*p* values were calculated using *t* statistic. SCL-90-R: Symptom Checklist-90 Revised; BFNE: Brief Fear of Negative Evaluation Scale; LSAS: Liebowitz Social Anxiety Scale; WHLS: Work, Home Management, Social and Private Leisure Activities Scale.

**Table 3:** Bivariate correlations among changes of symptom (*n* = 96).

	*Δ*SCL-90-R depression	*Δ*BFNE	*Δ*LSAS social	*Δ*LSAS public	*Δ*LSAS observation	*Δ*LSAS eating
*Δ*SCL-90-R depression	—	0.53^∗∗^	0.43^∗∗^	0.39^∗∗^	0.42^∗∗^	0.27^∗∗^
*Δ*BFNE		—	0.48^∗∗^	0.48^∗∗^	0.45^∗∗^	0.23^∗^
*Δ*LSAS social			—	0.64^∗∗^	0.69^∗∗^	0.46^∗∗^
*Δ*LSAS public				—	0.47^∗∗^	0.49^∗∗^
*Δ*LSAS observation					—	0.38^∗∗^
*Δ*LSAS eating						—

SCL-90-R: Symptom Checklist-90 Revised; BFNE: Brief Fear of Negative Evaluation Scale; LSAS: Liebowitz Social Anxiety Scale (^∗^*p* < 0.05, ^∗∗^*p* < 0.01).

**Table 4:** Predictors of WHLS work subscale changes (*n* = 96).

	Coefficient	Standardized coefficient	*t*	*p*
*Δ*SCL-90-R depression	0.32	0.13	1.15	0.25
*Δ*BFNE	0.01	0.07	0.57	0.57
*Δ*LSAS social interaction	0.02	0.14	0.93	0.35
*Δ*LSAS public speaking	0.12	0.35^∗∗^	2.69	<0.01
*Δ*LSAS observation by others	-0.02	-0.06	-0.42	0.67
*Δ*LSAS eating and drinking in public	-0.03	-0.04	-0.40	0.69

Adjusted *R*‐square = 0.21, *F* value = 5.18, degrees of freedom = 95, ^∗^*p* < 0.05, and ^∗∗^*p* < 0.01. SCL-90-R: Symptom Checklist-90 Revised; BFNE: Brief Fear of Negative Evaluation Scale; LSAS: Liebowitz Social Anxiety Scale; WHLS: Work, Home Management, Social and Private Leisure Activities Scale.

**Table 5:** Predictors of WHLS home management subscale changes (*n* = 96).

	Coefficient	Standardized coefficient	*t*	*p*
*Δ*SCL-90-R depression	0.53	0.23^∗^	1.99	<0.05
*Δ*BFNE	0.02	0.11	0.90	0.37
*Δ*LSAS social interaction	-0.01	-0.04	-0.23	0.82
*Δ*LSAS public speaking	0.01	0.03	0.19	0.85
*Δ*LSAS observation by others	0.03	0.08	0.61	0.54
*Δ*LSAS eating and drinking in public	0.12	0.17	1.45	0.15

Adjusted *R*‐square = 0.13, *F* value = 3.42, degrees of freedom = 95, ^∗^*p* < 0.05, and ^∗∗^*p* < 0.01. SCL-90-R: Symptom Checklist-90 Revised; BFNE: Brief Fear of Negative Evaluation Scale; LSAS: Liebowitz Social Anxiety Scale; WHLS: Work, Home Management, Social and Private Leisure Activities Scale.

**Table 6:** Predictors of WHLS social leisure activities subscale changes (*n* = 96).

	Coefficient	Standardized coefficient	*t*	*p*
*Δ*SCL-90-R depression	0.63	0.26^∗^	2.51	<0.05
*Δ*BFNE	0.00	0.10	0.10	0.92
*Δ*LSAS social interaction	0.04	0.30^∗^	2.20	<0.05
*Δ*LSAS public speaking	0.03	0.11	0.92	0.36
*Δ*LSAS observation by others	-0.03	-0.07	-0.61	0.54
*Δ*LSAS eating and drinking in public	0.12	0.17	1.66	0.10

Adjusted *R*‐square = 0.33, *F* value = 8.68, degrees of freedom = 95, ^∗^*p* < 0.05, and ^∗∗^*p* < 0.01. SCL-90-R: Symptom Checklist-90 Revised; BFNE: Brief Fear of Negative Evaluation Scale; LSAS: Liebowitz Social Anxiety Scale; WHLS: Work, Home Management, Social and Private Leisure Activities Scale.

**Table 7:** Predictors of WHLS private leisure activities subscale changes (*n* = 96).

	Coefficient	Standardized coefficient	*t*	*p*
*Δ*SCL-90-R depression	0.38	0.17	1.46	0.15
*Δ*BFNE	-0.02	-0.12	-0.96	0.34
*Δ*LSAS social interaction	-0.01	-0.06	-0.39	0.70
*Δ*LSAS public speaking	0.01	0.04	0.32	0.75
*Δ*LSAS observation by others	0.11	0.35^∗^	2.54	<0.05
*Δ*LSAS eating and drinking in public	0.06	0.09	0.80	0.43

Adjusted *R*‐square = 0.12, *F* value = 3.18, degrees of freedom = 95, ^∗^*p* < 0.05, and ^∗∗^*p* < 0.01. SCL-90-R: Symptom Checklist-90 Revised; BFNE: Brief Fear of Negative Evaluation Scale; LSAS: Liebowitz Social Anxiety Scale; WHLS: Work, Home Management, Social and Private Leisure Activities Scale.

## Data Availability

We also think that data sharing is important. However, the data in this study are not publicly available. Because this study was started in 2003, and in accordance with the protocol at that time, the study participants were informed that the data used in this study will not be provided to third parties other than the researchers and the participants.
